# Prematurity and the Risk of Development of Childhood Obesity: Piecing Together the Pathophysiological Puzzle. A Literature Review

**DOI:** 10.7759/cureus.20518

**Published:** 2021-12-19

**Authors:** Anupa Gnawali

**Affiliations:** 1 Family Medicine, University of Cape Town, Cape Town, ZAF

**Keywords:** prematurity, childhood obesity, noncommunicable disease, premature infants, preterm

## Abstract

One of the most devastating public health challenges in the twenty-first century is childhood obesity, and its prevalence is growing at a frightening rate. Premature infants have a greater likelihood of childhood obesity at age six to 16 compared to term infants. This study aims to explore the underlying mechanism of developing childhood obesity in this high-risk group. There are most likely multiple interconnected and supporting mechanisms that put this vulnerable population at risk of childhood obesity. Inflammation is a possible root cause. Prenatal causes included epigenetic changes as well as placental inflammation. Disturbances in hormonal pathways and elevated levels of serum bilirubin are possible explanations. Furthermore, preventable factors in the postnatal period were identified, such as weight gain and exclusive breastfeeding.

The prevalence of childhood obesity in preterm infants is high; thus, it is essential to understand the pathophysiology and address any preventable factors to decrease this disease burden.

## Introduction and background

Annually, 15 million babies are born prematurely, defined by the World Health Organization (WHO) as born before 37 completed gestation weeks. This number equates to more than one in 10 babies worldwide. Alarmingly, this number is rising. According to the WHO, the rate of preterm deliveries ranges from 5% to 18%. The prevalence is approximately 12% in lower-income countries and 9% in higher-income countries [1].

The multiple causes of premature births can be found in Table 1. Despite the advances in medical technology, which results in increased survival of preterm infants, many health problems are associated with this group of newborns. Problems include metabolic disease and obesity that continue well into adulthood [2].

**Table 1:** Causes of prematurity [1]

Maternal Origin	Fetal Origin	Placental Origin
Infection	Multiple gestations	Placental insufficiency
Chronic diseases: hypertension, diabetes	Congenital abnormalities	Abruptio placenta
Pre-eclampsia		Placenta previa
Socio-environmental: poor nutrition, smoking, alcohol, drugs, stress		

An extremely concerning public health challenge in the twenty-first century is childhood obesity, and its prevalence is growing at a frightening rate. In 2016, there were approximately 41 million obese or overweight children under five [3]. An international study highlighted that 10% of all school-aged children are overweight, and 25% are obese [4]. Being overweight and obese are pervasive and can lead to non-communicable diseases such as diabetes, metabolic, and cardiovascular disease at a younger age. Childhood obesity can also increase the risk of premature death and disability in adulthood [3]. Additionally, the morbidity of obesity-related conditions and medical treatment in later life contribute to high health care costs [5].

There is a higher prevalence of childhood obesity in preterm infants [2]. Thus, emphasizing the importance of understanding the preventable mechanisms that can be addressed to limit the spread of this rising public health crisis. There are many attributable factors to developing childhood obesity; however, this article will explore the link between premature infants and an increased prevalence of childhood obesity in this population.

Previous studies have found that obesity is a state of chronic low-grade inflammation [6]. A large study including 882 premature infants concluded that neonatal inflammation preceded the onset of obesity, suggesting that inflammation can contribute to the development of obesity [7]. The initiation of metabolic inflammation is thought to be as early as in the prenatal period, which can be influenced by multiple factors from either maternal or paternal environments [8].

**Figure 1:**
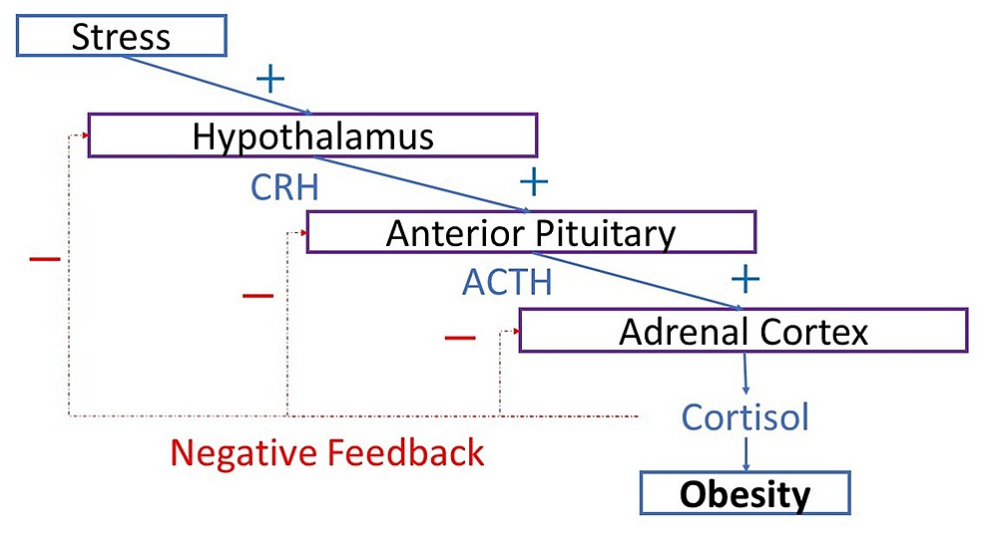
Hypothalamic pituitary adrenal axis dysfunction promoting obesity Any additional stress impacts the HPA axis and causes a rise in cortisol which promotes inflammation [9]. CRH: corticotropin-releasing hormone; ACTH: adrenocorticotropic hormone; HPA: hypothalamic-pituitary-adrenal

McEwen and Seeman emphasized that stress can alter the hypothalamic-pituitary-adrenal (HPA) axis (Figure 1). It is the primary mechanism that plays a role in adverse health consequences such as obesity in later life due to increased circulatory stress hormones such as cortisol [9].

A recent study done in 2019 explored the idea of elevated neonatal bilirubin levels as a proposed mechanism. Neonatal bilirubin levels had a positive trend of association with childhood obesity in preterm infants. However, the data collected was completed over 50 years ago and may not reflect the current trend of obesity and is the main limitation of this study [5]. 

Parental feeding patterns and introducing solid foods after six months of corrected gestational age are reported in some studies to potentially lower the risk of unfavoured weight trajectories into childhood [10]. This article will further explore feeding habits concerning accelerated weight gain or catch-up growth.

The review aims to highlight preventable mechanisms of childhood obesity in premature infants. Addressing these factors effectively will help decrease the burden of childhood obesity and its devastating complications. Childhood obesity is a worldwide burden, and this article can benefit both developed and developing countries.

## Review

Despite the rapid increase in prevalence and interest in childhood obesity over the last few years, the exact mechanism remains uncertain in the previously born premature population. The causes of childhood obesity can be examined through a developmental framework examining the role of metabolic inflammation. A host’s inflammatory response can be acute to remove the negative stimulus and return the body to the state before the injury. Chronic inflammation can be seen in which rapid clearance mechanisms fail, are incomplete, or in the case of obesity, where gradual or repeated alterations occur to normal physiology [8].

Many initiating events can cause the expansion of adipose tissue and trigger chronic systemic inflammation. These are seen in Table 2 and can be divided into prenatal, perinatal, and childhood factors. Prenatal factors are a programmed inheritance, including placental inflammation and epigenetic changes in paternal germ cells. Environmental factors include endotoxemia or microbiome alterations throughout the life course. Intrinsic growth rates are also crucial in the perinatal period as either intrauterine growth restriction or rapid growth. An essential factor in adipose tissue metabolism during adolescence and physical activity and dietary habits [8]. 

**Table 2:** The multiple causes of metabolic inflammation Throughout development, there are multiple events that can cause expansion of adipose tissue and cause systemic chronic inflammation [8].

Prenatal Factors	Perinatal Factors	Childhood Factors
Placental inflammation	Intra-uterine growth restriction	Inactivity/poor diet
Epigenetic changes in parental germ cells	Rapid growth/Catch-up growth	Impaired metabolism
	Endotoxemia/microbiome alterations	Endotoxemia/microbiome alterations
	Post-natal overfeeding adipose expansion/inflammation	Post-natal overfeeding adipose expansion/inflammation

A meta-analysis was done in 2020 examining prematurity and risk of childhood obesity showed that premature newborns had a higher likelihood of childhood obesity at the age of six to 16 compared to term infants [11]. No difference in childhood obesity was seen in preterm infants that were described as either small for gestational age (SGA) or appropriate for gestational age (AGA). Furthermore, prematurity increases the risk by 1.2 to develop a higher body mass index [11]. These findings were consistent with many previous studies done [12,13].

Prenatal factors

The Barker hypothesis and thrifty gene hypothesis claims that exposure in the prenatal and perinatal period increases the likelihood of preterm infants storing extra fat and energy and can increase the prevalence of chronic diseases later in life [11]. It suggests that inflammation can contribute to the development of obesity. The prenatal period can be influenced by both the maternal and paternal environment [8].

Animal studies revealed that paternal obesity also leads to epigenetic changes in sperm and triggers hypothalamic inflammation in the offspring [14]. Studies done in mice show that maternal obesity increases adipose tissue inflammation in the offspring. Placental inflammatory macrophages are elevated in obese mothers and release pro-inflammatory cytokines [15]. Maternal obesity is an additional risk factor for increased serum inflammatory markers in preterm infants and not in term newborns [16]. 

Researchers have also proposed that leptin plays a role in weight regulation in infancy and reports an association between higher leptin at age three and more significant weight gain and adiposity in later childhood [17]. 

The hypothalamic-pituitary-adrenal axis and its dysregulation in premature infants leading to obesity

The HPA axis is a primary mechanism thought to be closely linked to stress. Exposure to stress in utero and soon after delivery is high in premature infants. The initial maternal separation, the neonatal intensive care experience, and subsequent infections may have disastrous health and developmental challenges which affect the body’s stress response. It can also be observed in a dysregulation of the HPA axis [9]. Higher levels of HPA reactivity to stress early on in life and if experienced on a chronic basis throughout early childhood and adolescence are associated with adverse health consequences. This process results in higher levels of stress hormones such as cortisol, leading to cardiovascular and metabolic disease such as obesity, manifesting even in later childhood years or early adolescence [18]. 

Neonatal serum bilirubin in premature infants leading to obesity

Neonatal jaundice is common, and preterm infants are more susceptible to higher serum bilirubin levels than term newborns. As a result of the decreased survival of circulating fetal red blood cells, there is an increase in the bilirubin load in the hepatocytes. This results in the increased enterohepatic circulation of bilirubin. Additionally, there is a decrease in hepatic uptake of bilirubin in conjunction with defective bilirubin conjugation. As a result of increased neonatal red cell production and death, hepatic and gastrointestinal immaturity hyperbilirubinemia in preterm infants is more prevalent, severe and the duration is often protracted compared to term neonates [19]. 

A large prospective birth cohort was carried out over seven years, and the trend that was observed is higher the level of neonatal serum bilirubin, the higher the risk of childhood obesity [5]. The underlying mechanism suggested is that exposure to extreme levels of bilirubin results in neurotoxicity leading to more neurodevelopmental disabilities. Children with these disabilities often engage in less physical activity and have behavioral problems resulting in dysregulation of food intake and poor sleep. This results in a higher risk of developing childhood obesity [20].

However, this study has some limitations. The main one is that the data was collected more than 50 years ago: their birth weight, socioeconomic status, body composition, and maternal pregnancy factors are different from the preterm infants now. Additionally, genetic disorders which could contribute to jaundice and obesity were not differentiated. Caloric intake and exercise levels during childhood were not evaluated, which could be confounding factors. Multiple randomized trials need to be repeated to confirm if the relationship between bilirubin levels and childhood obesity is still relevant. 

Accelerated weight gain in premature infants leading to obesity

Aggressive nutritional intervention and catch-up growth in preterm infants were previously justified from a neurodevelopmental perspective. However, early weight gain may cause childhood obesity without advancing cognitive intelligence [11]. Weight is closely monitored in preterm infants, and there are usually strict discharge weight criteria. Thus, nutritional management for vulnerable preterm infants needs to be managed by a specialized team. Early nutritional consultation and a healthy catch-up to maintain a normal growth trajectory are key steps to preventing obesity [21].

A meta-analysis that compared 19 studies found that accelerated weight gain was a serious risk factor for developing childhood obesity. Accelerated weight gain significantly increased the risk of obesity by 2.69 times in children between eight to 11 years old [11]. This is a consistent finding, as other studies have shown that rapid postnatal growth in the first six to 12 months of life is a strong risk factor for metabolic disease [22].

Postnatal overfeeding has also been studied in rodents showing that it causes hypothalamic inflammation as early as 14 days after birth, leading to dysregulation of this axis even into adulthood. This suggests that the inflammatory set point of the brain is permanently changed by accelerated early life growth [23,24].

Feeding patterns in premature infants leading to obesity

Infancy (the first year of life) can be targeted as a critical period to prevent childhood obesity. Breastfeeding has been shown as a protective factor [25]. A meta-analysis showed that breastfeeding reduced the odds of being overweight and obese by 13% [26]. Another study found that each additional month of breastfeeding further reduced the prevalence of being overweight by 4% [27].

A higher prevalence of obesity is seen among all children who were never breastfed or who were breastfed for less than six months compared to those who were breastfed for more than six months-suggesting that breastfeeding is a preventative factor in developing childhood obesity [25]. Exclusively breastfeeding also prevents the early introduction of complementary foods that could lead to excessive weight gain. Studies have also shown that protein and total energy intake are lower in breastfed infants than formula-fed ones. Human milk is rich in *Bifidobacteria*, which is found to a lesser extent in obese children’s guts [28]. Furthermore, breast milk regulates food intake and energy balance due to the hormonal and biological factors it contains; this may help shape long-term physiological processes responsible for maintaining weight and preventing obesity. By promoting healthy weight gain in infancy, breastfeeding can potentially program an individual to lower the risk of obesity later in life [28]. 

Additionally, evidence shows that formula-fed infants have a higher plasma insulin level than breastfed infants, which stimulates fat deposition and early development of adipocytes [29]. Formula-fed neonates also tend to have higher body weight, which may suggest that both the higher protein intake and the weight gain in early life contribute to developing childhood obesity [30].

A large cohort study done in 2020 suggested that the most important predictor of childhood obesity is the trajectory of the body mass index z score (adjusted for the child’s age and sex). Introducing solid foods after six months of corrected gestational age could lower the risk of the unfavorable course of childhood obesity [10].

The postnatal period is a sensitive window for patterning both nutritional and inflammatory responses, which can correlate with long-lasting and devastating metabolic complications, such as obesity.

Limitations and recommendations

The study's main limitation is that risk factors such as socio-economic status of the families, complications at birth, and genetic disorders were not factored into all the studies selected and reported, which could negatively skew the results. Additionally, some studies used data collected from 50 years ago which may not still be relevant to today's population. 

Future recommendations include forming extensive sample-sized prospective birth cohort studies with a long follow-up period to accurately understand the underlying mechanisms that cause childhood obesity in the preterm population. Studies should also use standardized measurements detectable early in life, which can be repeated and measured later in childhood. By understanding the critical factors leading to childhood obesity, targeted management can help decrease the burden of obesity and metabolic comorbidities. 

## Conclusions

Premature infants are at increased risk of developing disastrous metabolic complications such as childhood obesity. The complex underlying mechanisms that can cause childhood obesity in infants born prematurely were explored. There are most likely multiple interconnected and supporting mechanisms that put this vulnerable population at risk of childhood obesity. Inflammation is explored as a possible root cause, along with prenatal factors, HPA axis dysregulation, and levels of neonatal serum bilirubin. Furthermore, preventable factors in the postnatal period were identified, such as weight gain and exclusive breastfeeding. Childhood obesity is increasing globally at an alarming rate, and this paper adds to the understanding of the critical factors that play a role in its mechanism in children born preterm. Future research needs to focus on creating long-term prospective cohort studies to find a temporal relationship between preterm infants and the causal link to childhood obesity. 
